# Retraction Note: Reprogramming glioblastoma multiforme cells into neurons by protein kinase inhibitors

**DOI:** 10.1186/s13046-021-02172-6

**Published:** 2021-11-18

**Authors:** Jie Yuan, Fan Zhang, Dennis Hallahan, Zhen Zhang, Liming He, Ling-Gang Wu, Meng You, Qin Yang

**Affiliations:** 1 Cancer Biology Division, Department of Radiation Oncology, Washington University School of Medicine, 4511 Forest Park, St. Louis, MO 63108 USA; 2 Medical Center of Stomatology, the First Affiliated Hospital of Jinan University, Guangzhou, 510630 China; 3 School of Stomatology, Jinan University, Guangzhou, 510630 China; 4 Synaptic Transmission Section, National Institute of Neurological Disorders and Stroke, Bethesda, MD 20892 USA

**Retraction Note: J Exp Clin Cancer Res 37, 181 (2018)**

**https://doi.org/10.1186/s13046-018-0857-5**

The Editor-in-Chief has retracted this article. An investigation by the Office of the Vice Chancellor for Research at Washington University in St. Louis concluded that:

- Figure panels 2a-f, labeled as the brain cancer cell line GBM U118, appeared to be falsified and/or fabricated, and were also used in multiple NIH grant applications as the brain cancer cell line GM139 or as the IMR90 cell line.
- Figure 3 appeared to be produced by cutting out and reusing three lanes from a heatmap that was previously used in a 2014 NIH grant application. The heatmap lanes in the grant application were labelled as iOPCs, WT (dermal fibroblasts) and OPC (Ctrl), while the lanes in Figure 3 in this publication were labeled as iN, GBM cells and Ctrl neuron.
- Figure 4c, labeled as iN cells in the 2018 this publication, appeared to be falsified and/or fabricated, and was also published in [1] labeled as iRLs from the breast cancer cell line MDA-MB-468.
- Figure panel 4d, labeled as GBM U18 cells, appeared to be produced by reusing, manipulating, and relabeling photographs from Figure 1c labeled as IMR90 cells in [2].
- An additional concern identified as part of the investigation is extensive overlap between this publication and [1].

In addition, the authors have been unable to provide any records of ethics approval for the animal study to the Editor. Limeng He and Zhen Zhang have stated that they were not aware of their inclusion in the author list of this study.

Dennis Hallahan, Zhen Zhang, Liming He and Ling-gang Wu agree to this retraction. Jie Yuan, Fan Zhang, Meng You and Qin Yang have not responded to any correspondence from the editor or publisher about this retraction.
